# Disparity in association of obesity measures with ankle and brachial systolic blood pressures in Europeans and South Asians

**DOI:** 10.1038/s41598-022-13372-1

**Published:** 2022-06-02

**Authors:** Matei Berceanu, Chew W. Cheng, Hema Viswambharan, Kirti Kain

**Affiliations:** 1 Leeds Institute of Cardiovascular and Metabolic Medicine, Faculty of Medicine and Health, University of Leeds, Leeds, LS2 9JT UK; 2 NHS England & NHS Improvement, North East and Yorkshire, Quarry Hill, Leeds, LS2 7UE UK

**Keywords:** Predictive markers, Biomarkers

## Abstract

Obesity causes increases in brachial systolic-blood-pressures (SBP), risks of type 2 diabetes (T2DM) and cardiovascular diseases (CVD). Brachial and ankle SBPs have differential relationship with T2DM and CVD. Our objective was to study the relationship of obesity measures with brachial and ankle SBPs. A population of 1098 adults (South Asians n = 699; 41.70% male and 58.3% female) were recruited over 5 years from primary care practices in England. Their four limbs SBPs were measured using Doppler machine and body-mass-index (BMI) and waist-to-height-ratio (WHtR) calculated. Linear regressions were performed between SBPs and obesity measures, after adjustments for sex, age, ethnicity, T2DM and CVD. The mean age of all participants was 51.3 (SD = 17.2), European was 57.7 (SD 17.2) and South Asian was 47.8 (SD = 16.1). The left posterior tibial [Beta = 1.179, *P* = 4.559 × 10^−15^] and the right posterior tibial SBP [Beta = 1.178, *P* = 1.114 × 10^−13^] most significantly associated with the BMI. In South Asians, although the left brachial [Beta = 25.775, *P* = 0.032] and right brachial SBP [Beta = 22.792, *P* = 0.045] were associated to the WHtR, the left posterior tibial SBP [Beta = 39.894, *P* = 0.023], association was the strongest. For the first time, we have demonstrated that ankle SBPs had significant association with generalised obesity than brachial systolic blood pressures (SBP), irrespective of ethnicity. However, with respect to visceral obesity, the association with ankle SBP was more significant in South Asians compared to Europeans.

## Introduction

Diabetes is a metabolic disorder that is estimated to affect 4.7 million people in the UK alone, as of 2020^1^. The current use of brachial blood pressures and the body mass index (BMI) in health screening programmes contributes over 50% of type 2 diabetes mellitus (T2DM) diagnoses, being missed^2^; a number estimated to be as high as 900,000 individuals in the UK^3^. This is due to the condition being subject to subclinical pathological mechanisms of insulin resistance. Insulin resistance leads up to the measurable increase in blood pressures or blood glucose or glycosylated haemoglobin, a critical period in which interventions would be most beneficial^4^ to prevent complications^5^. Early identification is essential, since late diagnoses add to the overall cost estimated to be 10% of the annual budget of the UK’s National Health System^6^.

Insulin resistance, a precursor of T2DM, has been found to cause perturbations of the arteriolar and capillary systems, that preferentially affect the lower limbs and not the upper limbs^7^. It is well-documented that ankle systolic blood pressure (SBP) can either be decreased by atherosclerotic narrowing in the femoral or popliteal arteries^8^ or increased by medial arterial wall stiffening and calcification^9,10^ which has been shown to correlate strongly to T2DM^11^. These findings have recently been highlighted, since ankle SBPs are independently associated with T2DM and cardiovascular diseases (CVD), more significantly than brachial SBPs^12^. However, the changes in ankle SBP have been studied mainly in the context of complications of peripheral arterial disease in older individuals, with brachial hypertension and type 2 diabetes. It is possible that in early stages of insulin resistance before the increase in blood glucose levels, ankle SBP would be affected before the brachial pressures. Therefore, these have been suggested as a more sensitive, yet equally inexpensive and alternative rapid screening tool.

A major risk factor contributing to the increasing incidence of T2DM is obesity, measured by the BMI with the values 18.5–25 kg/m^2^ being normal weight, 25–30 kg/m^2^ being overweight, and 30 + kg/m^2^ being obese^13^. The risk of diabetes increases three to seven-fold across the thresholds of BMI^14^ with CVD^15^. However, the BMI does not differentiate between lean mass and body fat, correlating less accurately with risks when considering women, the young, the elderly, or Asians and other ethnicities^16–18^. Visceral adiposity is measured by the waist-to-height ratio (WHtR)^19^, which is a better determinant of insulin resistance and undiagnosed diabetes^20^ than the BMI^21^. Due to both ankle SBPs and the WHtR being associated with insulin resistance, we hypothesised that ankle SBP will be strongly correlated to obesity parameters, especially the WHtR more than the brachial SBPs.

South Asians are more insulin resistant at younger ages at lower BMIs than Europeans^22^. The ankle blood pressure increase with type 2 diabetes is greater in South Asians compared to Europeans^23^. The primary outcome was to study the differences in the associations between the brachial and ankle SBP with BMI and WHtR. The secondary outcome: to study the ethnic differences in the above-mentioned associations.

## Results

### Characteristics of participants

**Figure 1:**
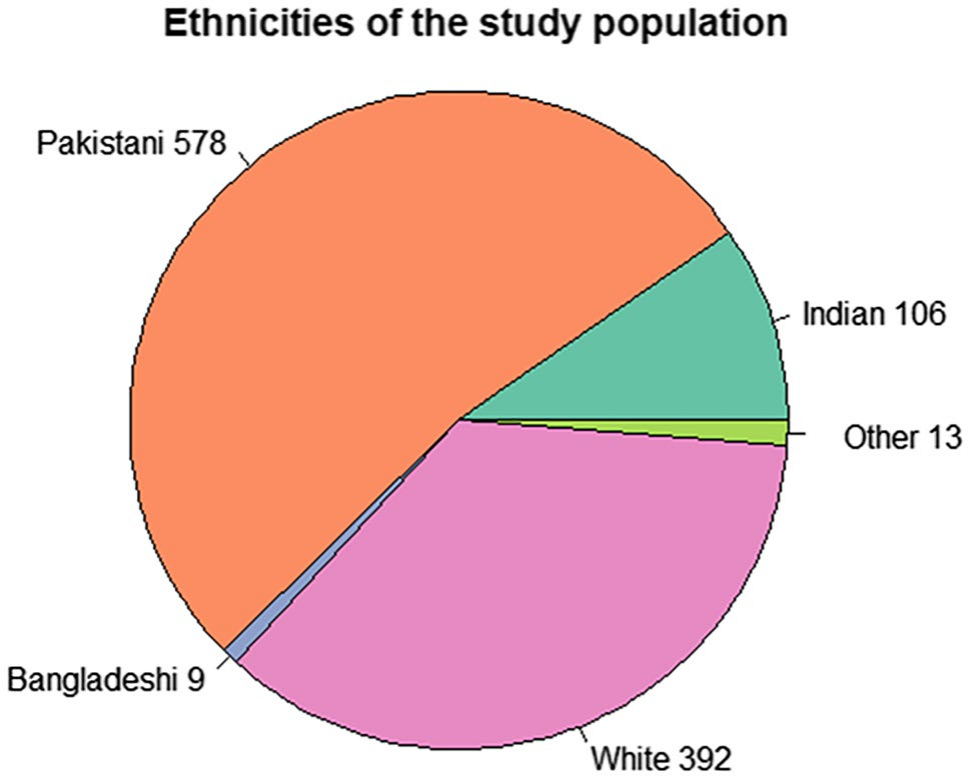
Ethnicities of the participants included in the statistical analyses of the study. Majority of the individuals are Pakistani and White Europeans, followed by Indian, and then Bangladeshi participants.

In total, 1098 patients were included in the statistical analyses of this study. Out of these, 392 were White Europeans, 699 were South Asian (made up of 578 Pakistani, 106 Indian, and 9 Bangladeshi) and 13 were from other ethnicities (Fig. 1). The general demographics, absolute SBPs, and clinical information of the population group are shown in Table 1. We found the South Asian cohort to be almost 10 years younger on average and to have a lower proportion of males compared to females (38.6% vs 48.0%, *P* = 0.0030). Despite there being no significant difference between the BMIs (28.9 kg/m^2^ in South Asians vs 29.2 kg/m^2^ in Europeans, *P* = 0.4064), the South Asians had a significantly higher WHtR (0.577 vs 0.558, *P* < 0.0001). Moreover, the prevalence of T2DM was significantly higher in South Asians (40.0% vs 30.9%, *P* < 0.01). However, they had a lower incidence of cardiovascular diseases (28.7% vs 38.8%, *P* < 0.001) compared to the White Europeans. Across the entire cohort, as well as across each subgroup, the average ankle SBPs were higher (range 139.7–157.6 mmHg) than the brachial ones (range 124.3–136.0 mmHg). However, all limb SBPs were significantly lower in the South Asian population (range 124.3–148.1 mmHg) than in the Europeans (range 131.6–157.6 mmHg, *P* < 0.0001).

**Table 1:** Demographics, limb systolic blood pressures, and clinical information regarding the entire cohort, also stratified by a European or south Asian subgroup.

Variables	Participants (1098)	European (392)	South Asian (700)	*P* value^†^
Age	51.3 (17.2)	57.7 (17.2)	47.8 (16.1)	< 0.0001*
Sex, % male	41.70%	48.00%	38.60%	0.003*^‡^
Body mass index, kg/m^2^	29.0 (6.2)	29.2 (6.0)	28.9 (6.3)	0.4064
Waist-to-height ratio	0.570 (0.070)	0.558 (0.066)	0.577 (0.071)	0.0001*
Right brachial, mmHg	130.0 (21.5)	136.0 (22.6)	126.7 (20.1)	< 0.0001*
Left brachial, mmHg	126.9 (19.7)	131.6 (20.8)	124.3 (18.5)	< 0.0001*
Right posterior tibial, mmHg	151.4 (32.6)	157.6 (35.3)	148.1 (30.4)	< 0.0001*
Left posterior tibial, mmHg	148.6 (30.6)	155.6 (33.6)	144.9 (28.2)	< 0.0001*
Right dorsalis pedis, mmHg	144.8 (31.2)	151.2 (33.8)	141.3 (29.1)	< 0.0001*
Left dorsalis pedis, mmHg	144.0 (31.3)	151.9 (32.9)	139.7 (29.5)	< 0.0001*
Cardiovascular disease	32.20%	38.80%	28.70%	0.001*^‡^
Type 2 diabetes mellitus	36.70%	30.90%	40.00%	0.003*^‡^
Antihypertensive treatment	44.17%	52.80%	39.71%	< 0.0001*^‡^

Data is presented as mean ± standard deviation (SD) or count (n).

*Statistically significant result using a P-value < 0.05.

^†^Comparison between European and South Asian subgroups.

^‡^*P* value calculated using a chi-squared test, rather than a T-test.

### Correlation between limb Systolic Blood Pressures and the Body Mass Index

Linear regressions were carried out to estimate the association between each individual limb SBP and the BMI (Table 2). All the limb SBPs were positively related to the BMI and the strongest associations were of the posterior tibial SBPs (*B* > 1.14, *P* < 0.001), followed by the dorsalis pedis SBPs (*B* > 0.82, *P* < 0.001), demonstrating that ankle SBPs were more strongly correlated to the BMI than brachial SBPs. In Model 1 (univariate), all SBPs were positively correlated with BMI (*B* = 0.534–1.209, *P* < 0.001). Therefore, 1-unit change in BMI, 0.594, 0.534, 1.150, 0.940, 1.209 and 0.872 mmHg high SBPs was observed. The left and the right posterior tibial SBPs were most significantly associated to the BMI (*P* < 0.001), followed by the left and the right dorsalis pedis SBPs (*P* < 0.001), and finally by the right and the left brachial SBPs (*P* < 0.001). The same order of significance remained constant throughout all models.

**Table 2:** Linear regressions comparing the individual systolic blood pressures with the waist to height ratio and the body mass index.

	Systolic blood pressure	Wait to height ratio			Body mass index		
		Coefficient	*P* value	Adjusted R	Coefficient	*P* value	Adjusted R
Model 1	Right brachial	60.877	**1.461 × 10**^**–9**^	0.038	0.594	**3.486 × 10**^−**7**^	0.027
	Left brachial	48.015	**8.889 × 10**^−**6**^	0.026	0.534	**1.400 × 10**^−**5**^	0.025
	Left posterior tibial	55.748	**1.568 × 10**^−**4**^	0.015	1.150	**5.124 × 10**^−**14**^	0.052
	Left dorsalis pedis	52.692	**3.909 × 10**^−**4**^	0.013	0.940	**1.806 × 10**^−**9**^	0.033
	Right posterior tibial	60.836	**8.562 × 10**^−**5**^	0.016	1.209	**7.099 × 10**^−**14**^	0.051
	Right dorsalis pedis	39.040	**8.669 × 10**^−**3**^	0.007	0.872	**1.836 × 10**^−**8**^	0.029
Model 2	Right brachial	26.967	**0.004**	0.245	0.490	**1.997 × 10**^−**6**^	0.266
	Left brachial	16.740	0.098	0.244	0.434	**6.560 × 10**^−**5**^	0.259
	Left posterior tibial	37.874	**0.012**	0.103	1.176	**1.474 × 10**^−**15**^	0.153
	Left dorsalis pedis	21.608	0.151	0.132	0.923	**3.809 × 10**^−**10**^	0.172
	Right posterior tibial	32.361	**0.042**	0.104	1.208	**6.791 × 10**^−**15**^	0.150
	Right dorsalis pedis	11.178	0.460	0.111	0.890	**2.121 × 10**^−**9**^	0.142
Model 3	Right brachial	27.75	**0.00**	0.26	0.51	**9.162 × 10**^−**7**^	0.27
	Left brachial	16.76	0.10	0.24	0.440	**5.868 × 10**^−**5**^	0.26
	Left posterior tibial	39.57	**0.01**	0.11	1.21	**2.744 × 10**^−**16**^	0.16
	Left dorsalis pedis	22.36	0.14	0.13	0.93	**2.747 × 10**^−**10**^	0.17
	Right posterior tibial	34.430	**0.030**	0.11	1.250	**1.116 × 10**^−**15**^	0.16
	Right dorsalis pedis	12.62	0.40	0.11	0.91	**8.619 × 10**^−**9**^	0.14
Model 4	Right brachial	26.11	**0.01**	0.25	0.48	**4.304 × 10**^−**6**^	0.26
	Left brachial	14.49	0.16	0.24	0.41	**2.093 × 10**^−**4**^	0.26
	Left posterior tibial	32.56	**0.03**	0.11	1.15	**1.854 × 10**^−**14**^	0.15
	Left dorsalis pedis	14.95	0.33	0.14	0.87	**8.888 × 10**^−**9**^	0.17
	Right posterior tibial	24.69	0.12	0.11	1.14	**6.176 × 10**^−**13**^	0.15
	Right dorsalis pedis	4.54	0.77	0.12	0.84	**3.819 × 10**^−**8**^	0.14
Model 5	Right brachial	26.51	**0.01**	0.25	0.50	**2.448 × 10**^−**6**^	0.27
	Left brachial	14.500	0.16	0.24	0.42	**1.882 × 10**^−**4**^	0.26
	Left posterior tibial	33.54	**0.03**	0.11	1.18	**4.559 × 10**^−**15**^	0.16
	Left dorsalis pedis	15.54	0.31	0.14	0.88	**6.387 × 10**^−**9**^	0.17
	Right posterior tibial	26.01	0.10	0.12	1.18	**1.114 × 10**^−**13**^	0.16
	Right dorsalis pedis	5.39	0.73	0.12	0.86	**1.926 × 10**^−**8**^	0.15
Model 6	Left brachial	0.48	0.90	0.25	0.40	**3.37 × 10**^−**4**^	0.27
	Right brachial	2.49	0.43	0.26	0.48	**3.74 × 10**^−**6**^	0.28
	Left posterior tibial	9.15	0.15	0.11	1.14	**4.18 × 10**^−**14**^	0.16
	Right posterior tibial	5.45	0.42	0.12	1.14	**7.41 × 10**^−**13**^	0.15
	Left dorsalis pedis	2.42	0.70	0.14	0.86	**1.40 × 10**^−**8**^	0.18
	Right dorsalis pedis	5.55	0.39	0.13	0.82	**7.83 × 10**^−**8**^	0.15

Model 1: univariate analysis.

Model 2: adjusted for sex, age, and ethnicity.

Model 3: adjusted for sex, age, ethnicity, CVD.

Model 4: adjusted for sex, age, ethnicity, T2DM.

Model 5: adjusted for sex, age, ethnicity, CVD, T2DM.

Model 6: adjusted for age, sex, ethnicity, cardiovascular diseases, and Type 2 diabetes, and hypertension treatments.

Bold text: statistical significance using a *P* value < 0.05.

CVD: cardiovascular disease; T2DM: type 2 diabetes mellitus.

Model 2 revealed significant positive associations of SBPs to BMI when adjusted for sex, age, and ethnicity (*B* = 0.434–1.208, *P* < 0.001). BMI was significantly positively associated with increased SBPs. More specifically, the BMI was significantly associated with 1.176 and 1.208 mmHg higher in left and the right posterior tibial SBPs.

In Model 3 (adjusted for sex, age, ethnicity, CVD), the BMI was strongly associated to the left and the right posterior tibial SBPs (*B* = 1.21 and 1.250, *P* < 0.001), followed by the left and the right dorsalis pedis SBPs (*B* = 0.93 and 0.91, *P* < 0.001), and finally by the right and the left brachial SBPs (*B* = 0.51 and 0.440, *P* < 0.001). In Model 4 (adjusted for sex, age, ethnicity, and T2DM), the left and the right posterior tibial SBPs were significantly associated to the BMI (*B* = 1.15 and 1.14, *P* < 0.001), followed by the left and the right dorsalis pedis SBPs (*B* = 0.87 and 0.84, *P* < 0.001), and by the right and the left brachial SBPs (*B* = 0.48 and 0.41, *P* < 0.001).

In Model 5 (adjusted for sex, age, ethnicity, CVD, and T2DM), the association of BMI was higher in the left and the right posterior tibial SBPs (*B* = 1.18, *P* < 0.001). Moreover, the relationship of BMI to right and the left brachial SBPs had a trend towards lower association (*B* = 0.50, 0.42, *P* < 0.001).

In Model 6 (adjusted for sex, age, ethnicity, CVD, T2DM, and hypertension treatments), BMI showed significant and very strong positive correlations with the left and the right posterior tibial SBPs (*B* = 1.14, *P* < 0.001), followed by the left and the right dorsalis pedis SBPs (*B* = 0.86 and 0.82, *P* < 0.001), and finally by the right and the left brachial SBPs (*B* = 0.40 and 0.48, *P* < 0.001).

### Correlation between limb systolic blood pressures and the waist height ratio

**Figure 2:**
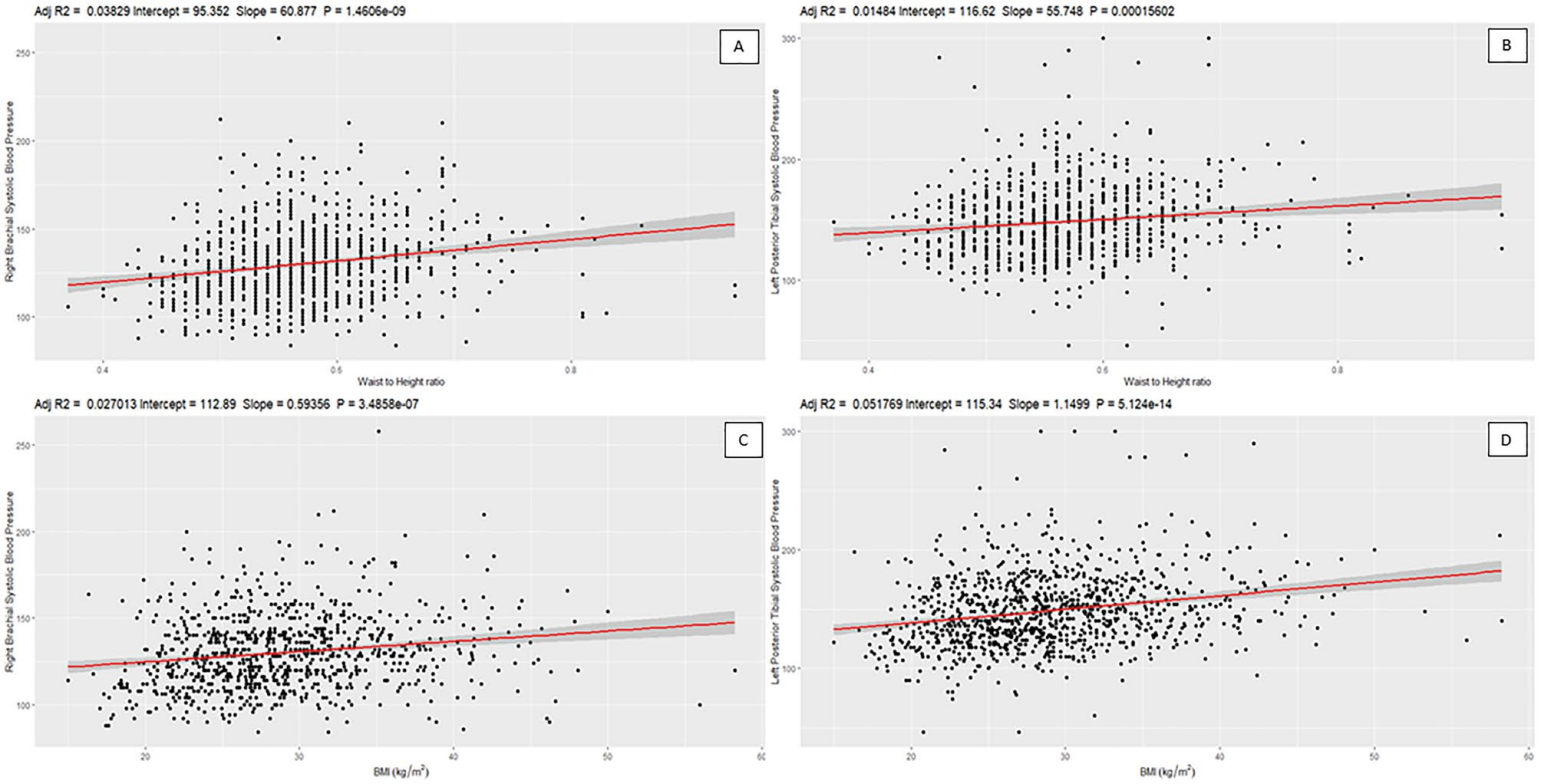
Four examples of linear regressions used. The top panels show the correlation between the right brachial (**A**) and the left posterior tibial (**B**) against the waist to height ratio. The bottom panels show the correlation between the right brachial (**C**) and the left posterior tibial (**D**) against the body mass index (BMI). Each black dot represents an individual result, with the red line showing the line of best fit and the grey area surrounding this showing a small 95% confidence interval of where the true line lies. On each graph, the adjusted R2 value is shown, along with the y-intercept, the slope gradient, and the *P* value of the association. These two specific systolic blood pressures were chosen as an example due to their statistical significance in all models.

Using the same six models, linear regressions were also carried out between each individual limb SBP and the WHtR (Table 2). The results indicated that the difference in SBPs were evaluated for 1-unit change in waist-to-height ratio. In the univariate model, all limb SBPs showed significant positively associations (*B* = 39.04–60.877, *P* < 0.001), with the strongest associations observed in the right brachial and the right posterior tibial, respectively (*B* = 60.877, 60.836, *P* < 0.001). The lowest association was found in the right dorsalis pedis (*B* = 39.040, *P* < 0.001). In models 2 and 3, we found significant associations in the right brachial (*B* = 26.967, 27.75, *P* < 0.01), the left posterior tibial (*B* = 37.874, 39.57, *P* < 0.05) and right posterior tibial (*B* = 32.361, 34.430, *P* < 0.05) SBPs to WHtR. We did not find a correlation between other SBPs and WHtR. Models 4 and 5 revealed two significant positive correlations in the right brachial and left posterior tibial SBPs to the WHtR. The strongest association was observed in the left posterior tibial (*B* = 32.56, 33.54, *P* < 0.05), followed by right brachial (*B* = 26.11, 26.51, *P* < 0.05). In Model 6, we did not detect any significant associations of all SBPs to the WHtR, albeit stronger associations were observed amongst ABPs. While, limb SBPs were not as strongly correlated to the WHtR as they were to the BMI, the ankle SBPs demonstrated slightly weaker associations compared to the brachial SBPs. Four examples of the correlation graphs are illustrated in Fig. 2, which showed similar trends for brachial and ankle SBP, with each being correlated against the WHtR, as well as the BMI.

### Ethnicity-specific associations between limb Systolic Blood Pressures and Obesity parameters

The participants were grouped into either Europeans or South Asians (Pakistani, Indian, Bangladeshi and other South Asian ethnicities) (Table 3). This model was adjusted for sex, age, CVD, and T2DM. All individual pressures in both groups were significantly correlated to the BMI (*P* < 0.05), with the left posterior tibial SBP (*P* < 0.001) and the right posterior tibial SBP (*P* < 0.001) consistently having the stronger significant positive associations, regardless of ethnicity. In terms of the WHtR, it was only the left posterior tibial SBP (*P* < 0.05), the left brachial SBP (*P* < 0.05), and the right brachial SBP (*P* < 0.05) in South Asians that had significant associations. In Europeans, none of the SBPs had significant relationships with the WHtR. When compared to brachial SBPs, ankle SBPs were more strongly associated to the WHtR in South Asians, as well as to the BMI regardless of ethnicity.

**Table 3:** Linear regressions comparing the different systolic blood pressures measured against either the waist to height ratio or BMI by ethnic group.

Systolic blood pressure	Ethnicity	WHtR				BMI			
		n	Coefficient	*P* value	Adjusted R	n	Coefficient	*P* value	Adjusted R
Left brachial	European	250	-15.436	0.439	0.194	250	0.516	**0.012**	0.212
	South Asian	460	25.775	**0.032**	0.241	461	0.348	**0.009**	0.245
Right brachial	European	331	27.150	0.140	0.188	330	0.762	**< 0.001**	0.218
	South Asian	581	22.792	**0.045**	0.244	582	0.350	**0.004**	0.249
Left posterior tibial	European	316	18.349	0.549	0.052	365	1.249	**< 0.001**	0.096
	South Asian	574	39.894	**0.023**	0.116	678	1.112	**< 0.001**	0.157
Right posterior tibial	European	327	12.268	0.698	0.046	375	1.411	**< 0.001**	0.102
	South Asian	573	25.684	0.161	0.148	676	1.026	**< 0.001**	0.168
Left dorsalis pedis	European	322	-9.554	0.752	0.093	369	0.776	**0.007**	0.122
	South Asian	578	26.700	0.134	0.132	672	0.912	**< 0.001**	0.154
Right dorsalis pedis	European	326	-37.654	0.206	0.069	371	0.609	**0.038**	0.079
	South Asian	576	22.917	0.194	0.143	671	0.959	**< 0.001**	0.162

Model is adjusted for adjusted for sex, age, cardiovascular disease, and type 2 diabetes mellitus.

The ethnicities have been grouped into either European or south Asian (composed of Indian, Pakistani Bangladeshi, and other south Asian ethnicities).

## Discussion

To our knowledge, this is the first ever study investigating specifically, the associations between absolute ankle SBP and obesity parameters. We have shown ankle SBP to be independently associated with the BMI. We have also shown a stronger independent association between the left posterior tibial SBP and the WHtR when compared to brachial SBPs in South Asians.

Brachial SBP have been shown to correlate to the BMI^24^. However, there is very little information regarding ankle SBP.

Other studies have previously used the ankle brachial index (ABI), measured by the ratio of ankle SBP to the brachial SBP and studied its relationship to the BMI. A large prospective study of different ethnicities showed a positive correlation between BMI and ABI^25^. Moreover, a different study with a similar dataset demonstrated a high baseline BMI is independently and positively associated with an increase in the ABI^26^. Several other studies have also shown positive correlations between the BMI and the ABI: an epidemiological study from Italy^27^, another involving individuals at high risk of CVD or with prevalent CVD^28^, and two studies regarding participants with either metabolic syndrome^29^ or diabetes^30^. However, a prospective study of individuals without CVD showed no correlation between high ABI and BMI, although participants with low ABIs were excluded^31^. We are aware that the ABI as an index, and its value can therefore, rise either due to an increase in the ankle SBP or a decrease in the brachial SBP. However, among the studies mentioned above, the difference in the brachial SBPs between the normal and high ABI groups was either not significant^25^, or the participants in the high ABI group had higher brachial SBP^28,30,31^. As a result, it was an increase in the ankle SBP relative to the brachial SBP that resulted in the higher ABI, making the results comparable to our study. Overall, there is indeed a positive correlation between the high ABI (and therefore, ankle SBPs) and the BMI, with this association being seemingly strengthened in the context of CVD and/or T2DM.

Our results are in agreement with the few studies that have used absolute ankle SBP. In a small sample of Brazilian participants, the average SBP of the participants’ posterior tibial arteries and brachial arteries increased with their BMIs^32^. Moreover, two large prospective studies found that individuals with higher ankle SBP also had significantly higher BMIs^33,34^. These studies further strengthened the evidence of the association between ankle SBP and BMIs. Interestingly, our study demonstrated the left and the right posterior tibial SBP to be more significantly associated with the BMI than other pedal or brachial SBPs. Changes in the BMI in lean individuals have previously been shown to have less of an effect on their brachial SBP, compared to BMI changes in the overweight or obese^35^.

The metabolic changes associated with insulin resistance are more pronounced in the lower extremities^7,36^. This is further demonstrated by atherothrombotic occlusive changes, preferentially affecting lower limbs resulting in higher rates of lower limb amputations, with the upper limbs rarely being subject to adverse pathological changes^37^. Since the WHtR has been found to be the best indicator of insulin resistance^21^ and undiagnosed T2DM^20^, we hypothesised that ankle SBP would be more significantly related to the WHtR than brachial SBP. However, we found only the right brachial SBP and the left posterior tibial SBP had a significant relationship with the WHtR, with the former association being the most significant. It seems that the SBP does not correlate with the WHtR, as strongly as it does with the BMI, regardless of whether it is measured from the arms or the legs. Since the study done on the whole study population, a possible explanation might be that visceral obesity, leading to insulin resistance, T2DM and the resultant increase in ankle SBP and CVD are more critical in only certain ethnicities.

To our knowledge, there are very few studies investigating the direct relationship between absolute ankle SBPs and the WHtR. Our findings reflect previous results which showed a significant correlation between the BMI and the ABI, compared to central obesity and the ABI^26^. However, central obesity was measured using the waist circumference and not the WHtR. Other studies have found a moderate positive association between the WHtR and the ABI, with a high WHtR also being a significant predictor of a high ABI, even after adjusting for several cardiovascular, metabolic and renal factors^30^. Despite the ABI being used and not the absolute ankle SBP, the study concluded that it was the diffuse changes, specifically affecting the vessels of the lower limbs that resulted in the high ABI, and not a change in the brachial SBP.

Other researchers have reported conflicting results, with one study on Chinese elderly showing a positive correlation between the WHtR and the ABI in the healthy group, but not one between the BMI and the ABI^38^. Interestingly, in a Ghanaian population, a high ABI was found to be negatively correlated to both the WHtR and the BMI^39^. However, all participants had peripheral arterial disease which lowers the ankle blood pressure and could therefore, explain these findings. Moreover, diabetes can cause both an increase and decrease of ABP, depending on the stage of the diabetes and insulin resistance. It is possible that the ABP increases in earlier stages of insulin resistance due to the changes in the local microcirculation of lower limbs and in the later stages, of arterial wall stiffening. However, the ABP could decrease in diabetes because of the narrowing of lower limb arteries in the later stages. Overall, the literature is inconclusive of the association between ankle SBP and the WHtR, although it points to a positive correlation. ABI is a ratio which is usually measured to assess occlusive peripheral arterial disease or arterial stiffness of lower limbs as a complication of ageing, hypertension or type 2 diabetes. The high ABI could be due to increased ankle blood pressures or lower brachial blood pressures. Therefore, it is difficult to interpret differential associations of obesity measures with ankle blood pressures versus brachial. For the above reason, we did not analyse the data in relation to ABI.

Since blood pressure is systemic, a change would congruently affect the measurements, regardless of where they are taken from, albeit to a different magnitude. Therefore, it can be deduced that ankle SBP, which we found to be moderately and positively correlated to brachial SBP would associate with different measures of obesity, equally. However, our results show more significant correlation between ankle SBP and BMI, rather than WHtR. This raises the question of whether the obesity parameter’s strength at measuring actual visceral fat differs between the ethnicities. Specific ethnicities would therefore, show a significant correlation between the limb SBP and obesity parameters, depending on whether the BMI or the WHtR was used.

In South Asian and Europeans, both groups showed all limb SBPs to be significantly associated with the BMI. The posterior tibial artery SBP consistently was most significantly correlated with the BMI, both in the whole study sample, as well as individual South Asians and Europeans groups, demonstrating that the evidence is more conclusive of its association to the BMI, regardless of ethnicity.

With respect to the WHtR, we found the two brachial SBPs, as well as the left posterior tibial SBP, to show statistical significance in South Asians only. It is important to note that, the left posterior tibial SBP had the most significant association, further highlighting the potential of increased ankle SBPs reflecting levels of adiposity. South Asians have more cardio-metabolic risk factors at younger ages which manifest at lower weights than Europeans. They also have more visceral fat at the same BMI as Europeans^40^. It is for this very reason why South Asians’ BMI thresholds were revised and updated, and that the WHtR has been shown to measure their actual visceral fat more accurately than the BMI^41,42^. South Asians’ increased risk at lower ages is reflected in our study, where despite our sample being purposely enriched in T2DM with a significantly higher number of South Asians having T2DM, they were almost 10 years younger on average than the Europeans. Moreover, all their limb SBPs were significantly lower and their BMIs were not statistically different; results which would be unexpected in the context of higher T2DM prevalence. The only parameter indicative of this would be the WHtR, which we found to be significantly higher in South Asians, possibly due to its stronger association to visceral fat and insulin resistance. This might explain the reason some of the limb SBP were significantly correlated with the WHtR, only in South Asians. Similarly, it could also explain the negative coefficients found in Europeans which show that as the WHtR increases, the SBP tends to decrease; an unexpected negative association not found in any of the analyses involving the BMI. Overall, it implicated that the use of ankle SBPs and the WHtR might be more beneficial in South Asians than in Europeans. Since the left posterior tibial SBP correlates best with both the BMI in the general population and with the WHtR in South Asians, it could be the one out of the four ankle SBPs that could be used in practice.

Our findings have very important implications. Many diagnoses of T2DM are missed at screening^2^. Since ankle SBPs being more significantly associated to generalised obesity, their values would increase more consistently with obesity compared to brachial SBPs, thus identifying more individuals at risk of developing T2DM or CVD^12^. Similarly, the use of ankle SBPs would be especially useful in South Asians, as their values would be more consistently affected by the WHtR. As these individuals tend to have brachial SBPs within the currently recognised normal thresholds and more visceral fat per BMI than Europeans, the use of ankle SBPs with the WHtR might prove to be more suitable in identifying risks of T2DM and CVD^12^.

Increased ankle SBP as a surrogate of insulin resistance along with BMI threshold in the first trimester might prove to be useful in predicting gestational diabetes in third trimester, especially in a primigravida without a family history of T2DM.

ABP are more significantly associated with diabetes than brachial blood pressure^12^. However, in this study we focused only on the relationship of ABP with obesity measures. Building on the basis of the results we have published and in this study, ankle SBP is a tentatively better than brachial SBP as an effective screening tool.

## Strengths and limitations

This is first ever study that investigated the relationship of absolute ankle SBP with obesity parameters. Moreover, the large sample size makes the results more reliable and significant.

There are several limitations to this study: Firstly, we used a cross-sectional design to investigate the correlation between ankle SBP and obesity parameters. The causality, therefore cannot be inferred, further warranting longitudinal studies to confirm the associations found, since visceral adiposity may be more important to South Asians. This is because they, generally demonstrate higher adiposity. Moreover, our study sample is not representative of the general population in the community, as they were recruited in primary care clinics and we did not have socioeconomic status data to use in the analysis.

## Conclusion

We have demonstrated that ankle SBP (more specifically the posterior tibial SBPs) are significantly associated to the BMI. In South Asians, ankle SBP was significantly associated with WHtR. Further prospective clinical and scientific studies are needed to confirm these results.

## Methods

The project was approved by the Local Research Ethics Committee (REC 10/H1302/28) and local Research and Development. All methods and experimental protocols were carried out in accordance with the Declaration of Helsinki^43^. In addition, all methods and experimental protocols were reviewed and approved by a body equivalent to the present 2016 Integrated Research Application System UK. Written informed consent was obtained from each participant according to Good Clinical Practice guidelines^12,44^.

The methods employed by this study have been described, previously^44^. The study uses cross-sectional data that was obtained during the 2010–2014 period at an inner-city primary care practice in West Yorkshire. This was performed in accordance with the Strengthening the Reporting of Observational Studies in Epidemiology guidelines^45^. Only adults (18 years of age or older) were included in the study. Patients were excluded if they were undergoing chemotherapy or if they were too ill to participate. Ethnicity was determined based on medical records or was self-reported by the participants at recruitment (having at least one grandparent born in those regions). There were six different ethnic groups, but most were of South Asian or European descent. Suitable participants were approached and provided with information regarding the study and with the chance to ask any questions. Those who agreed were asked for written informed consent^12,44^.

### Clinical assessment

The primary care clinic uses an electronic database containing details regarding diagnoses, procedures, patient attendances, consultations and costs^44^. This database also includes patient information gathered from pharmacies, laboratories, hospitalisations, or outpatient diagnoses. A detailed questionnaire was used, with the information being validated against primary care records. Any of the following conditions were classified as CVD: myocardial infarction or heart failure, stroke or transient ischaemic attack, peripheral arterial disease, angioplasty, and coronary artery bypass surgery. Participants with a diagnosis of T2DM on basis of standard laboratory blood tests were identified through the use of medical records or through self-reporting^44^.

All clinical assessments were carried out at the same visit^44^. Prior to the blood pressures being measured, the patients rested for a period of 5 min in the supine position. A single measure of blood pressures were then measured using an appropriately sized cuff based on participants’ arm size and a Doppler instrument (Huntleigh Super Dopplex II, Huntleigh Healthcare, Cardiff, UK). For the brachial SBP, the cuff selected based on the size of participants, was placed on the upper arm, with the Doppler probe being placed in the antecubital fossa over the brachial artery in each arm. For the ankle SBP, the cuff was placed superior to the medial malleolus, with the probe measuring the pressures of the dorsalis pedis and the posterior tibial in both legs. The cuffs were inflated to 20 mmHg above the approximate pressures at which the pulse disappeared and slowly deflated until the pulse was audible again, with the value being recorded. For the BMI (kg/m^2^) calculations, the participant’s heights and weights were recorded to the nearest 0.01 m and 0.01 kg, respectively. For the WHtR, the waist circumference was measured to the nearest 0.01 m at the midpoint between the iliac crest and the lowest rib. The recordings were done by trained researchers using the same equipment, thus reducing inter-instrumental errors^44^.

### Statistical analyses

The data was compiled in Excel and analysed using RStudio (Version 1.4.1103, RStudio, PBC) and STATA (Version 16.1 for Windows, College Station, Texas 77,845 USA). Linear regressions were carried out to examine the association of the six SBP measures, two brachial, two dorsalis pedis, and two posterior tibial (dependent variables) with the BMI or the WHtR (independent variables). Five models were made, progressively adjusting for more covariates: Model 1: univariate analysis; Model 2: adjusted for sex, age, and ethnicity; Model 3: adjusted for sex, age, ethnicity, CVD; Model 4: adjusted for sex, age, ethnicity, T2DM; Model 5: adjusted for sex, age, ethnicity, CVD, T2DM. Model 6: adjusted for sex, age, ethnicity, CVD, T2DM and treatment for hypertension. Due to the small number of participants in certain ethnicities, a general cohort encompassing South Asians (consisting of Pakistani, Indian, Bangladeshi and other South Asian participants) was created. This was compared to the European cohort through a series of t-tests and chi squared tests (performed for sex, CVD, or T2DM). In any statistical analyses, P values lower than 0.05 were considered as being significant using a 95% confidence interval.

## Data Availability

The datasets generated during and/or analysed during the current study are not publicly available since patient permission was not sought for the sharing of data, at the time of recruitment.

## References

[1] NCVIN. Diabetes Prevalence Model for England + estimated growth between 2015–2020 from AHPO (2010) Prevalence Models for Scotland and Wales. (2016).

[2] Casagrande SS, Cowie CC, Fradkin JE (2013). Utility of the U.S. Preventive Services Task Force criteria for diabetes screening. Am. J. Prevent. Med..

[3] NCVIN. (2016).

[4] Purnell, J. Q. in *Endotext* (eds K. R. Feingold *et al.*) (MDText.com, Inc, 2000).

[5] DiabetesUK. *Us, diabetes and a lot of facts and stats*, <https://www.diabetes.org.uk/resources-s3/2019-02/1362B_Facts%20and%20stats%20Update%20Jan%202019_LOW%20RES_EXTERNAL.pdf> (2019).

[6] Hex N, Bartlett C, Wright D, Taylor M, Varley D (2012). Estimating the current and future costs of Type 1 and Type 2 diabetes in the UK, including direct health costs and indirect societal and productivity costs. Diabet Med.

[7] Hile C, Veves A (2003). Diabetic neuropathy and microcirculation. Curr. Diab. Rep..

[8] London GM, Pannier B (2010). Arterial functions: How to interpret the complex physiology. Nephrol. Dial. Transplant..

[9] Young MJ, Adams JE, Anderson GF, Boulton AJ, Cavanagh PR (1993). Medial arterial calcification in the feet of diabetic patients and matched non-diabetic control subjects. Diabetologia.

[10] Everhart JE, Pettitt DJ, Knowler WC, Rose FA, Bennett PH (1988). Medial arterial calcification and its association with mortality and complications of diabetes. Diabetologia.

[11] Aboyans V (2008). The association between elevated ankle systolic pressures and peripheral occlusive arterial disease in diabetic and nondiabetic subjects. J. Vasc. Surg..

[12] Viswambharan H, Cheng CW, Kain K (2021). Differential associations of ankle and brachial blood pressures with diabetes and cardiovascular diseases: cross-sectional study. Sci. Rep..

[13] Nuttall FQ (2015). Body Mass Index: Obesity, BMI, and health: A critical review. Nutr. Today.

[14] PublicHealthEngland. *Adult obesity and type 2 diabetes*, <https://assets.publishing.service.gov.uk/government/uploads/system/uploads/attachment_data/file/338934/Adult_obesity_and_type_2_diabetes_.pdf> (2014).

[15] WHO. *Obesity and overweight Fact Sheet*, <https://www.who.int/news-room/fact-sheets/detail/obesity-and-overweight> (2020).

[16] Shai I (2006). Ethnicity, obesity, and risk of type 2 diabetes in women: a 20-year follow-up study. Diabetes Care.

[17] Wells JC (2007). Sexual dimorphism of body composition. Best Pract Res Clin Endocrinol Metab.

[18] de Onis M, Habicht JP (1996). Anthropometric reference data for international use: recommendations from a World Health Organization Expert Committee. Am. J. Clin. Nutr..

[19] Ashwell M, Gibson S (2016). Waist-to-height ratio as an indicator of 'early health risk': simpler and more predictive than using a 'matrix' based on BMI and waist circumference. BMJ Open.

[20] Xu Z, Qi X, Dahl AK, Xu W (2013). Waist-to-height ratio is the best indicator for undiagnosed type 2 diabetes. Diabet Med..

[21] Jamar G (2017). Evaluation of waist-to-height ratio as a predictor of insulin resistance in non-diabetic obese individuals. A cross-sectional study. Sao Paulo Med. J..

[22] Gujral UP, Pradeepa R, Weber MB, Narayan KM, Mohan V (2013). Type 2 diabetes in South Asians: Similarities and differences with white Caucasian and other populations. Ann. N. Y. Acad. Sci..

[23] Kain K (2013). Ankle pressures in UK South Asians with diabetes mellitus: A case control study. Heart.

[24] Staessen J, Fagard R, Amery A (1988). The relationship between body weight and blood pressure. J. Hum. Hypertens..

[25] Criqui MH (2010). The ankle-brachial index and incident cardiovascular events in the MESA (Multi-Ethnic Study of Atherosclerosis). J. Am. Coll. Cardiol..

[26] Tison GH (2011). Usefulness of baseline obesity to predict development of a high ankle brachial index (from the Multi-Ethnic Study of Atherosclerosis). Am. J. Cardiol..

[27] Signorelli SS (2011). Prevalence of high ankle–brachial index (ABI) in general population of Southern Italy, risk factor profiles and systemic cardiovascular co-morbidity: An epidemiological study. Arch. Gerontol. Geriatr..

[28] Hendriks Eva JE (2016). Association of high ankle brachial index with incident cardiovascular disease and mortality in a high-risk population. Arteriosclerosis Thrombosis Vasc. Biol..

[29] Zhang Y (2013). Inflammation and oxidative stress are associated with the prevalence of high aankle-brachial index in metabolic syndrome patients without chronic renal failure. Int. J. Med. Sci..

[30] Depczynski B, Young T, White C (2018). A high ankle-brachial index is associated with obesity and low serum 25-hydroxyvitamin D in patients with diabetes. J. Clin. Transl. Endocrinol..

[31] Velescu A (2017). Abnormally high ankle-brachial index is associated with all-cause and cardiovascular mortality: The REGICOR study. Eur. J. Vasc. Endovasc. Surg..

[32] Freitas D (2014). Cardiovascular risk in white coat hypertension: An evaluation of the ankle brachial index. J. Vasc. Nurs..

[33] Hietanen H, Pääkkönen R, Salomaa V (2008). Ankle blood pressure as a predictor of total and cardiovascular mortality. BMC Cardiovasc. Disord..

[34] Hietanen H, Pääkkönen R, Salomaa V (2010). Ankle and exercise blood pressures as predictors of coronary morbidity and mortality in a prospective follow-up study. J. Hum. Hypertens..

[35] Jones DW, Kim JS, Andrew ME, Kim SJ, Hong YP (1994). Body mass index and blood pressure in Korean men and women: The Korean National Blood Pressure Survey. J. Hypertens..

[36] Britton KA (2012). Insulin resistance and incident peripheral artery disease in the Cardiovascular Health Study. Vasc Med.

[37] Marso SP, Hiatt WR (2006). Peripheral arterial disease in patients with diabetes. J. Am. Coll. Cardiol..

[38] Liu Y (2014). Correlation between anthropometric parameters and arteriosclerosis biomarker in the middle-aged and the elderly. Beijing Da Xue Xue Bao Yi Xue Ban.

[39] Yeboah K (2016). Body composition and ankle-brachial index in Ghanaians with asymptomatic peripheral arterial disease in a tertiary hospital. BMC Obes..

[40] Misra A, Khurana L (2009). The metabolic syndrome in South Asians: Epidemiology, determinants, and prevention. Metab. Syndr. Relat. Disord..

[41] Jayawardana R, Ranasinghe P, Sheriff MH, Matthews DR, Katulanda P (2013). Waist to height ratio: A better anthropometric marker of diabetes and cardio-metabolic risks in South Asian adults. Diabetes Res Clin Pract.

[42] Prasad D, Kabir Z, Suganthy J, Dash A, Das B (2013). Appropriate anthropometric indices to identify cardiometabolic risk in South Asians. WHO South-East Asia J. Public Health.

[43] World Medical A (2013). World Medical Association Declaration of Helsinki: Ethical principles for medical research involving human subjects. JAMA.

[44] Kain K (2013). Ankle pressures in UK South Asians with diabetes mellitus: A case control study. Heart (British Cardiac Society).

[45] STROBE statement--checklist of items that should be included in reports of observational studies (STROBE initiative). *Int J Public Health* **53**, 3–4. 10.1007/s00038-007-0239-9 (2008). 10.1007/s00038-007-0239-9 18522360

